# Supplementary material for: Assessing an Outdoor Office Work Intervention: Exploring the Relevance of Measuring Frequency, Perceived Stress, Quality of Life and Connectedness to Nature
Source: Healthcare (Basel). 2025 Jul 11;13(14):1677. doi: 10.3390/healthcare13141677 (PMC12294554; doi:10.3390/healthcare13141677)
Supplement: Supplementary file 1 [file healthcare-13-01677-s001.zip › healthcare-3647952-supplementary.pdf]

TIDieR

Pop-out outdoor office work programme

Why:

Support employees, at small- and medium-sized workplaces, in transitioning relevant everyday office tasks outdoors.

What (material):

The intervention group at the workplaces followed a course of activities divided into four main activity-cluster lead by facilitators.

What (procedures):

The first activity-cluster consisted of prepping an ambassador at each workplace. The role of the ambassador was to take lead of the intervention and with it followed the possibility of receiving support from the facilitators every other week. Furthermore a group profile with information on the intervention was set up on LinkedIn for every participant in the intervention group to follow.

The second activity-cluster followed the first one and started out with two tutorials, of 10 min. each, for the participants to watch giving them information about the intervention: "*Experiences From Sweden*" and *"Pop-out – Get Off to a Good Start Working Outdoors."* Two weeks after followed by a kick-off session of 45 min. that included a presentation of the benefits of everyday office tasks outdoors. Bases on an environmental assessment of the outdoor setting at each workplace pictures of different types of settings and routs were presented as well as examples of tasks to take out and the facilities this would require. Following the presentation a poster with pictures of the different types of outdoor settings and routes specific to each workplace was put up as a tool for the participants to mark and share their activities outdoors. Furthermore the participants were asked to conduct an exercise selecting one or two office tasks to take out during the first week of the intervention. This was supported by a list of potential office tasks that could be performed outdoors.

The third activity-cluster was initiated a month after the second activity-cluster by two tutorials of 5 min each motivating participants for everyday office tasks outdoors: *"Why Is It Challenging to Go Out? And Where Should We Go?"* and "*To persist - from experience to good habits".* This was followed by a webinar of 45 min. encouraging participants to share their experiences of everyday office tasks outdoors.

The fourth activity-cluster concluded the course of activities in a workshop after the 12-week long intervention. The workshop focused on gathering experiences and motivate the participants to continue their outdoor tasks. At the end of the workshop a website was introduced giving access to information about everyday office tasks outdoors including the tutorials that were part of the intervention plus three new ones: "What Do I Gain from Working Outdoors?"; "What Work Tasks Can I Take Outside with Me?" and "Get Started Working Outdoors".

See also *article section 2.2. The Intervention and* Supplementary Materials, Figure S1 : Intervention Activities

Who provided:

The facilitators of the intervention were experts within the field of workplace mental health, management, and organizational studies. Information on the outdoor setting at each of the workplaces part of the kick-off session in the second activity-cluster was provided by an expert within landscape architecture.

Pop-out outdoor office work programme

**How (mode of delivery; individual or group):**	The information to the participants of the intervention was given through their ambassador, the group profile on LinkedIn, e-mails, sessions at the workplaces, a printed poster and list of examples of tasks, webinars, workshops at the workplaces and a website
**Where:**	The intervention took place at the workplaces where the participants use different types of outdoor settings and routes of own choice in the vicinity of the workplaces. The types of outdoor setting varied from workplace to workplace and included terraces, courtyards, gardens, parks, streetscape greenery, harbour areas, beaches, bush lands and forests.
**When and how much:**	The intervention had a duration of 12 weeks between March and June 2022 starting with second activity-cluster and ending with the fourth activity-cluster. The third activity-cluster was initiated a month after the second activity-cluster.
**Tailoring:**	Participants all followed the same intervention but the activities part of it were voluntary.
**How well (planned):**	The number of participants taking part of the intervention was registered but the attendance in each specific activity part of the activity-clusters was not registered.
